# Physical activity and liver disease affect the fat-free mass in adolescents with cystic fibrosis

**DOI:** 10.1007/s00431-022-04752-w

**Published:** 2022-12-07

**Authors:** Theodore Dassios, Maria Rafaela Mitakidou, Anil Dhawan, Niovi Papalexopoulou, Atul Gupta, Anne Greenough

**Affiliations:** 1 Department of Women and Children’s Health, School of Life Course Sciences, Faculty of Life Sciences and Medicine, King’s College London, London, UK; 2 Neonatal Intensive Care Centre, King’s College Hospital NHS Foundation Trust, 4th Floor Golden Jubilee Wing, London, SE5 9RS UK; 3 Pediatric Liver, GI & Nutrition Centre and Mowat Labs, King’s College Hospital, London, UK; 4 Institute of Liver Studies, King’s College London, London, UK; 5 Pediatric Respiratory Medicine, Kings College Hospital NHS Foundation Trust, London, UK; 6 Asthma UK Centre in Allergic Mechanisms of Asthma, King’s College London, London, UK; 7 NIHR Biomedical Research Centre at Guy’s and St Thomas’ NHS Foundation Trust and King’s College London, London, UK

**Keywords:** Body composition, Adolescent, Cystic fibrosis, Liver diseases

## Abstract

Cystic fibrosis (CF) is predominantly a lung disease but is also characterised by impaired skeletal muscularity and a reduction in fat-free mass. We aimed to test the hypothesis that clinical and anthropometric parameters would determine fat-free mass impairment in adolescents with CF. We measured the fat-free mass index (FFMI) using bioelectrical impedance, the lung function using spirometry, the number of shuttles as a measure of exercise tolerance and the reported physical activity in children and young people with CF in a tertiary centre at King’s College Hospital, London, UK. CF-related liver disease was diagnosed by abnormal liver enzymes and/or ultrasonography. We studied 28 children and young people (11 male) with a median (interquartile range (IQR)) age of 15 (13–17) years. They had a median (IQR) FFMI of 13.5 (11.6–15.1) kg/m^2^. The FFMI significantly correlated with age (rho = 0.568, *p* = 0.002), number of shuttles (rho = 0.691, *p* < 0.001) and reported hours of activity per day (rho = 0.426, *p* = 0.024). The median (IQR) FFMI was significantly higher in male [15.1 (13.1–18.6) kg/m^2^] compared to female participants [12.7 (11.6–14.1) kg/m^2^, *p* = 0.008]. The median (IQR) FFMI was significantly lower in the 10 (36%) participants with liver disease [11.9 (11.5–13.4) kg/m^2^] compared to the FFMI in the remaining 18 participants without liver disease [14.4 (12.5–15.9) kg/m^2^, *p* = 0.027].

*Conclusion*: Fat-free mass increases with increasing age and growth in adolescents with CF. Physical activity exerts a beneficial effect on fat-free mass, and CF-related liver disease negatively affects fat-free mass in adolescents with CF.

**Table:** 

**What is Known:** *• Health behaviours in adolescence influence lifelong health in cystic fibrosis (CF).* *• A normal body mass index in CF might fail to reveal a low fat-free mass (FFM), and quality of life in CF is strongly associated with a reduced FFM.*	
**What is New:** *• FFM increases with increasing age and growth in adolescents with CF. * *• Physical activity exerts a beneficial effect, and liver disease negatively affects FFM in adolescents with CF.*

**What is Known:**

*• Health behaviours in adolescence influence lifelong health in cystic fibrosis (CF).*

*• A normal body mass index in CF might fail to reveal a low fat-free mass (FFM), and quality of life in CF is strongly associated with a reduced FFM.*

**What is New:**

*• FFM increases with increasing age and growth in adolescents with CF. *

*• Physical activity exerts a beneficial effect, and liver disease negatively affects FFM in adolescents with CF.*

## Introduction

Cystic fibrosis (CF) is a life-shortening, hereditary disease with patients commonly suffering from frequent lung infections and obstructive pulmonary disease. Nutritional impairment is common in CF and is typically due to a chronic negative energy balance secondary to malabsorption [1]. Monitoring of weight, height and the corresponding body mass index (BMI) are frequently used in clinical practice to support optimal pulmonary function in children and adolescents, with a recommendation to maintain a BMI above the fiftieth percentile [2]. The BMI, however, cannot inform on body composition which is known to be globally affected in CF, including a decreased fat-free mass which corresponds to decreased skeletal muscularity and muscle atrophy [3]. A seemingly normal BMI might fail to reveal a low fat-free mass [4], and a high BMI could mask a significantly reduced muscle mass [5] in the context of overweight or obesity which is becoming increasingly common in the CF population [6].

Peripheral muscle abnormalities go often hand-in-hand with respiratory muscle impairment [7] and are thought to be the combined result of different pathophysiological processes, such as physical inactivity, chronic inflammation, and the frequent use of agents that cause muscle atrophy such as systemic corticosteroids [8].The cystic fibrosis transmembrane conductance regulator is also expressed in human skeletal muscle, leaving open a possibility of an intrinsic primary effect of the disease on the skeletal muscles [9]. Reduced muscle mass is of critical clinical importance in CF as outcomes such as lung function, quality of life and mortality have been more strongly associated with a reduced fat-free mass rather than with a low body mass index [8].

New health behaviours appear during adolescence, which track into adulthood and influence lifelong health and morbidity. Poor adherence to treatment in adolescence is often considered to be a major contributor to the decline in CF lung health [10]. The determinants of fat-free mass have not been previously investigated in adolescents with CF. We hypothesised that clinical and anthropometric parameters would be associated with the fat-free mass in adolescents with CF. Our aim was to determine these parameters.

## Methods and materials

### Study design

This study was a secondary analysis of a study cohort which aimed to validate results from a bioelectrical impedance device against dual-energy X-ray absorptiometry (DEXA) [11]. The study investigated clinically stable children and young people with CF aged between 12 and 19 years. The study was conducted between June 2016 and January 2017 at the tertiary CF centre at King’s College Hospital National Health System Foundation Trust, London, UK [11]. Consecutive children and young people were enrolled. Any children or young people with a pulmonary exacerbation in the past 2 weeks or acute illness and hospitalisation were excluded from the study. Age, gender and genotype were recorded. The genotype was classified as homozygous for ΔF508, heterozygous for ΔF508 or other mutations. Information on the presence of chronic infection (three or more positive microbiology sputum cultures in the preceding 12 months) [11], CF-related diabetes, CF-related liver disease (abnormal liver enzymes and/or ultrasonography) [12] and pancreatic insufficiency, concurrent use of systemic steroids and 5-year survival was recorded. The participants underwent bioelectrical impedance analysis, pulmonary function and exercise tolerance tests. Ethical approval was granted by the East Midlands—Nottingham II Research Ethics Committee (reference: 16/EM/0174). Participants aged 16 years and older gave written consent, whilst participants younger than 16 years gave written assent, and consent was provided by their parents/guardians.

### Fat-free mass and body mass index

The fat-free mass was measured by bioelectrical impedance analysis [13] with the InBody S10 Body Composition Analyser (InBody Ltd, Cerritos, CA, USA). Fat-free mass was estimated by impedance using the four compartment model representing the body in terms of water, protein, fat and mineral components. Measurements were taken using the tetra-polar 8-Point Tactile Electrode system in the sitting position, following 2 h of fasting and micturition within 30 min prior to testing. The fat-free mass was adjusted using a correction equation that was derived following regression with DEXA as gold standard and was specific for this bioelectric impedance device [11]. The fat-free mass index (FFMI) was calculated as fat-free mass/height^2^ (kg/m^2^) [14]. Height was measured to the nearest cm by a stadiometer and weight to the nearest 0.1 kg by digital scales. The corresponding BMI *z*-score and percentile were calculated [15].

### Pulmonary function and respiratory muscle testing

Spirometry and body plethysmography were performed on the same day with bioelectrical impedance analysis, according to the American Thoracic Society (ATS) and the European Respiratory Society (ERS) guidelines [16, 17]. The Jaeger MasterScreen PFT/IOS/Body, CareFusion Ltd, Basingstoke, UK, was used to measure pulmonary function. The highest value of forced vital capacity (FVC), forced expiratory volume in 1 s (FEV_1_) and functional residual capacity (FRC) was recorded and was expressed in *z*-scores [18]. Maximal inspiratory pressure and maximal expiratory pressure were measured and expressed as percentage predicted [19]. Maximal inspiratory and maximal expiratory pressures were measured using a handheld, manometer according to ATS/ERS guidelines (Micro RPM Respiratory Muscle Analyser, CareFusion, San Diego, CA, USA) [20].

### Habitual activity estimation scale

Participants completed the Habitual Activity Estimation Scale on the day of the assessment. The parents and/or members of the research team assisted as required. They reported the percentage of an average day that they were inactive (lying down), somewhat inactive (sitting), somewhat active (walking) and active (activities that make you breathe hard and increase your heart rate). They documented their waking, sleeping and meal times. The research team was thus able to calculate the hours spent on each of these activity levels. The hours of being active and somewhat active were then summed to calculate the time spent being moderately-vigorously active per day.

### Modified shuttle walk test

The modified shuttle walk test was used to assess exercise tolerance. Patients walked/run between two cones, on an enclosed flat corridor with externally paced instructions. Initial walking speed was 0.50 m/s and was increased by 0.17 m/s every minute until the participant completed all 15 levels or became too breathless to maintain the required speed or failed to complete the shuttle in the allocated time [21].

### Statistical analyses

Continuous data were tested for normality with the Kolmogorov–Smirnov test, were found to be non-normally distributed and were presented as median and interquartile range (IQR). The relationship between the FFMI and pulmonary function, respiratory muscle function and exercise variables were examined using Spearman’s rho bivariate correlation analysis (rho). The FFMI was compared in binary conditions such as sex, chronic infection, CF-related diabetes, pancreatic insufficiency and CF-related liver disease using the chi-square test. To examine the independent association of the fat-free mass with pulmonary function, exercise and clinical parameters, the continuous parameters that exhibited a significant correlation with the FFMI and the binary parameters in which the FFMI was significantly different (*p* < 0.05) were inserted in a multivariable linear regression model with the FFMI as the outcome variable. Multi-collinearity among the independent variables in the regression analysis was assessed by examination of a correlation matrix for the independent variables. Univariate linear regression analysis was used to graphically depict the relationship of the FFMI with the number of shuttles. Statistical analysis was performed using SPSS software, version 27.0 (SPSS Inc., Chicago, IL, USA).

## Results

**Fig. 1:**
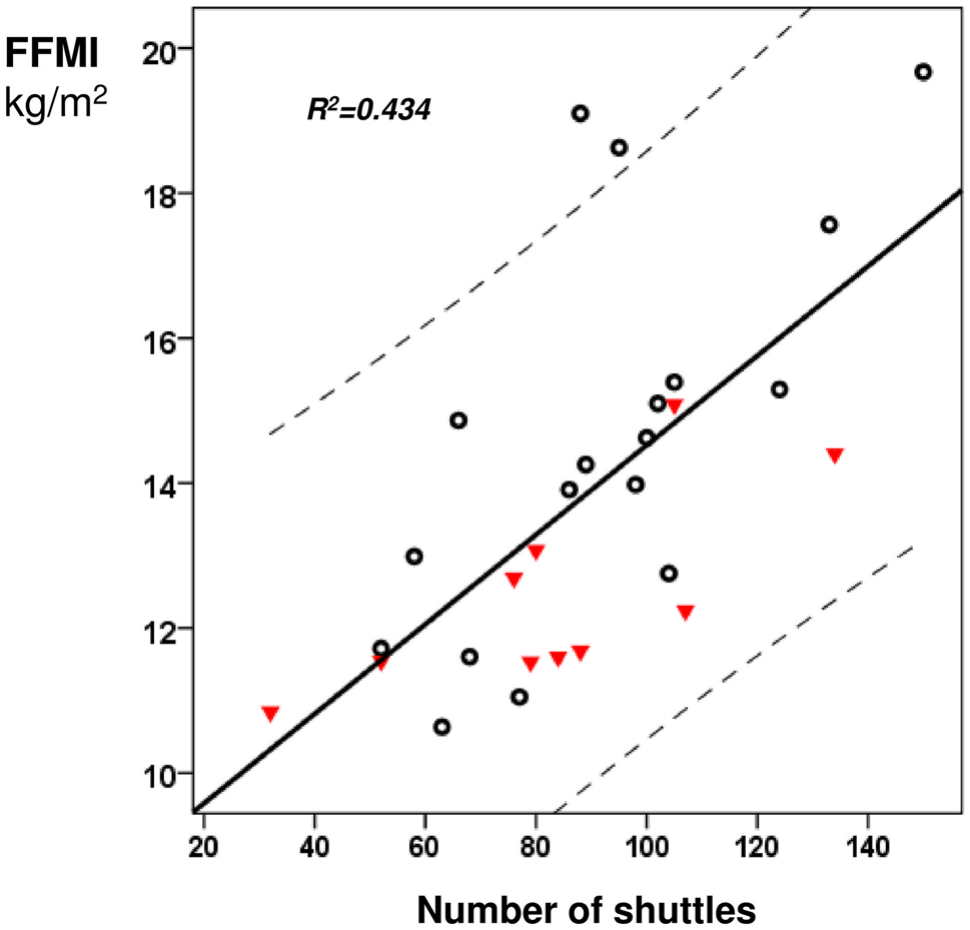
Univariate linear regression of the FFMI with the number of shuttles. The subjects with CF-related liver disease are presented as inverted triangles and the subjects without CF-related liver disease as unfilled circles. The regression line and 95% confidence intervals are presented

**Fig. 2:**
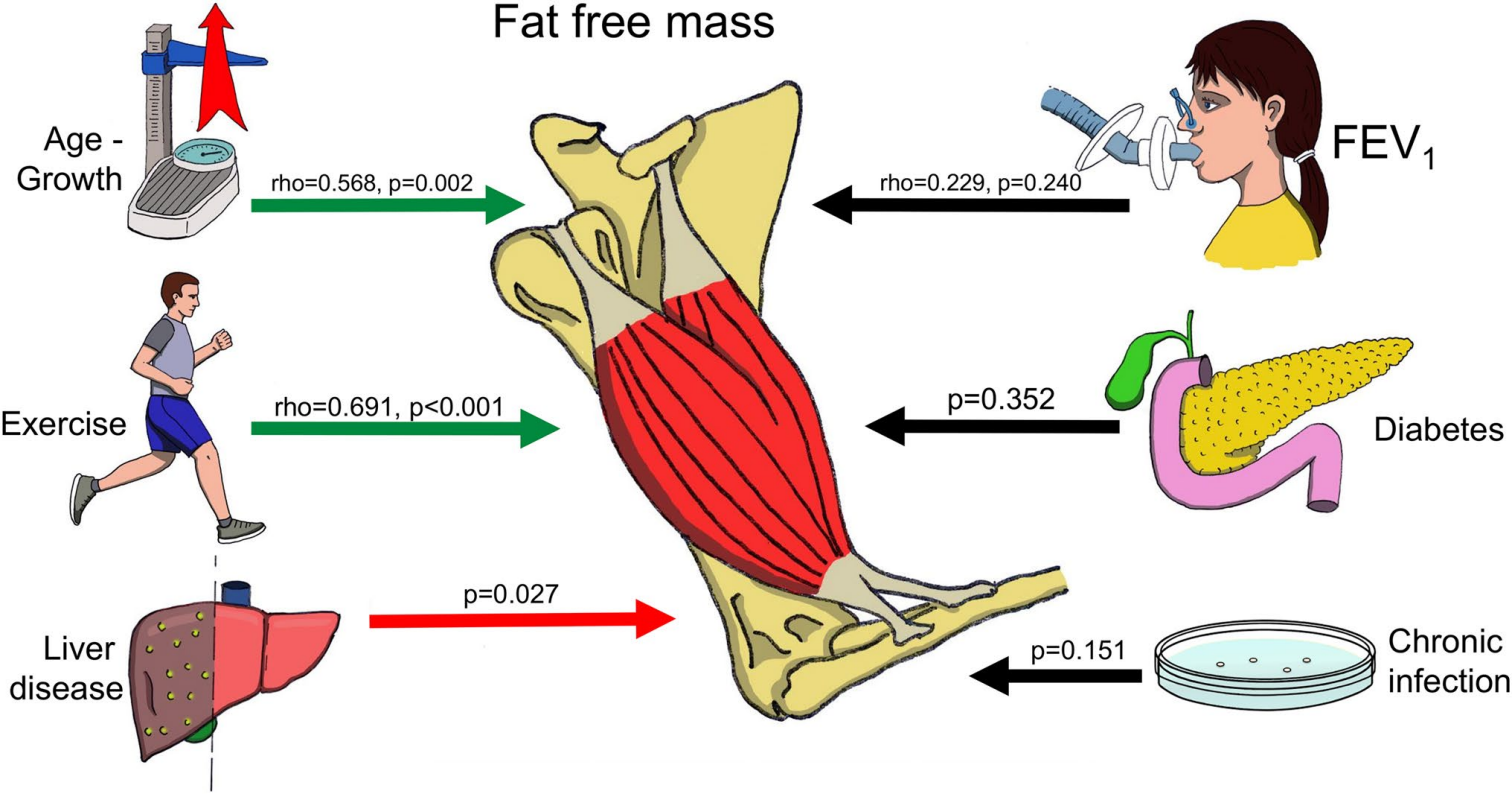
Graphical summary of the significant (left) and non-significant (right) associations of fat-free mass with the included clinical and demographic parameters

During the study period, 28 children and young people (11 male) were recruited to the study with a median (IQR) age of 15 (12–19) years. One subject that was recruited in the primary study was excluded from this study as fat-free mass could not be measured as part of the original data collection. The demographic and clinical characteristics of the included subjects are presented in Table 1. They had a median (IQR) fat-free mass of 37.7 (30.9–47.4) kg and a FFMI of 13.5 (11.6–15.1) kg/m^2^. The FFMI significantly correlated with age (rho = 0.568, *p* = 0.002), number of shuttles (rho = 0.691, *p* < 0.001, Fig. 1), reported hours of activity per day (rho = 0.426, *p* = 0.024), FVC *z*-score (rho = 0.388, *p* = 0.041), maximum inspiratory (rho = 0.394, p = 0.038) and maximum expiratory (rho = 0.493, *p* = 0.008) pressure but not with BMI *z*-score (rho = 0.178, *p* = 0.364), FEV_1_ *z*-score (rho = 0.229, *p* = 0.240) and FRC *z*-score (rho =  − 0.299, *p* = 0.156). The median (IQR) FFMI was significantly higher in male [15.1 (13.1–18.6) kg/m^2^] compared to female participants [12.7 (11.6–14.1) kg/m^2^, *p* = 0.008] and significantly lower in participants with liver disease [11.9 (11.5–13.4) kg/m^2^] compared to participants without liver disease [14.4 (12.5–15.9) kg/m^2^, *p* = 0.027, Fig. 1]. The ultrasonographic findings and liver function tests of the subjects with liver disease are presented in Table 2. The median (IQR) FFMI was not different in patients with chronic infection [12.2 (11.5–14.9) kg/m^2^] compared to patients without chronic infection [13.9 (12.8–15.3) kg/m^2^, *p* = 0.151]. The median (IQR) FFMI was not different in participants with CF-related diabetes [12.2 (11.3–14.5) kg/m^2^] compared to participants without diabetes [14.0 (11.6–15.3) kg/m^2^, *p* = 0.352]. The FFMI was not compared in participants with and without pancreatic insufficiency as there was only one subject without pancreatic insufficiency. A summary of the associations of the fat-free mass with the included clinical and anthropometric parameters is graphically presented in Fig. 2.

**Table 1:** Patient demographics and clinical characteristics. Data presented as median (IQR) or *N* (%)

*N*	28
Age (years)	15 (13–17)
Weight (kg)	50.2 (44.4–56.7)
Height (m)	1.57 (1.51–1.67)
Male	11 (39)
Genotype	
ΔF508 homozygous	16 (57)
ΔF508 heterozygous	9 (32)
Other CFTR mutation	3 (11)
Pancreatic insufficiency	27 (96)
Chronic infection	17 (61)
CF-related liver disease	10 (36)
CF-related diabetes	5 (18)
Concurrent use of systemic steroids	1 (4)
BMI *z*-score	− 0.02 (− 0.75–0.59)
FFM (kg)	37.7 (30.9–47.4)
FFMI (kg/m^2^)	13.5 (11.6–15.1)
FEV_1_ *z*-score	− 1.80 (− 2.80 to − 0.94)
FVC *z*-score	− 0.96 (− 1.46–0.04)
FRC *z*-score	0.94 (− 0.06–3.03)
MEP % predicted	99.7 (87.7–117.6)
MIP % predicted	105.8 (84.6–124.4)
Shuttles	88 (70–105)
Moderate to vigorous activity (hrs/week)	11.3 (8.1–14.4)
5-year survival	27 (96)
Days of intravenous antibiotics	28 (14–53)

*CFTR* cystic fibrosis transmembrane conductance regulator, *BMI* body mass index, *FFMI* fat-free mass index, *FEV*_*1*_ forced expiratory volume in 1 s, *FVC* forced vital capacity, *FRC* functional residual capacity, *MEP* maximal expiratory pressure, *MIP* maximal inspiratory pressure

**Table 2:** Ultrasonographic findings and liver function tests in the group of CF-related liver disease. Data presented as median (range) or *N* (%)

*N*	10 (36)
**Ultrasound**	
Fatty liver	8 (80)
Splenomegaly	5 (50)
**Laboratory**	
AST (units/L)	34 (17–128)
GGT (units/L)	22 (3–143)
Bilirubin (μmol/L)	8 (2–11)
Platelets (10^9^/L)	254 (56–418)
APRI	0.123 (0.069–2.286)

*AST* aspartate aminotransferase, *GGT* gamma-glutamyl transferase, *APRI* AST to platelet ratio

Weight and age were not included in the multivariable model due to collinearity with height, since the FFMI is a product of the square of height. The hours of activity per week were collinear with the number of shuttles and were thus not included in the model. Following multivariable linear regression analysis, the fat-free mass was independently associated with male sex (adjusted *p* = 0.028, 95% CI = 0.19 to 3.07), number of shuttles (adjusted *p* = 0.004, 95% CI = 0.02 to 0.08) and CF-related liver disease (adjusted *p* = 0.033, 95% CI =  − 2.91 to − 0.14) but not with the FVC *z*-score (adjusted *p* = 0.773, 95% CI =  − 0.63 to 0.48).

## Discussion

We have demonstrated that fat-free mass increases with increasing age and growth in adolescents with cystic fibrosis and that the fat-free mass is positively affected by aerobic activity and negatively affected by the development of CF-related liver disease. Our hypothesis that clinical and anthropometric parameters would be associated with the fat-free mass in adolescents with CF was thus accepted.

Our population is clinically comparable to previous studies of body composition in CF. Scully and colleagues studied 38 adolescents and adults with CF at a mean age of 28 years with a mean FEV_1_ of 73% predicted and reported a mean lean mass of 42.2 kg measured by DEXA [22]. In our study, we measured a younger population at a median age of 15 years and reported a median FEV_1_ of 77.3% predicted and a FFMI of 37.7 kg. The actual values of the fat-free mass in our subjects are, as expected, lower compared to healthy children and young people of a similar age [22]. In our study, we report a median fat-free mass of 37.7 kg, while population studies in healthy 13–18 year old children have reported mean fat-free mass values of 38.9–41.8 kg [23]. The finding of a more reduced fat-free mass in CF patients with liver disease is a novel finding in the whole CF population and not only in CF adolescents. The precise mechanism underlying how liver disease can induce muscle atrophy has not been completely elucidated. A recent animal study described a potential mechanism where liver fibrosis (induced in mice by bile duct ligation) leads to the upregulation of the atrophy-inducing, tumour necrosis factor α in a culture of human myotubes [24]. It is interesting to note that focal biliary fibrosis is the main pathological process in CF-related liver disease [12]. Furthermore, liver disease is associated with a hypermetabolic state, increased energy requirements and a reduction in caloric intake and absorption. These phenomena might also explain the skeletal muscle atrophy seen with liver disease. Kyrana et al. compared the fat-free mass measured by DEXA in seventeen children with end-stage chronic liver disease (none of them with CF) at a median age of 7.4 years to 14 healthy controls and reported that resting energy expenditure correlated strongly with fat-free mass, but the ratio of the resting energy expenditure to the fat-free mass was not different between patients and controls [25].

The finding of a higher fat-free mass and improved muscularity in adolescents with higher reported physical activity and exercise capacity would be an expected finding as active individuals would have a higher muscle mass. Our study did not include an exercise intervention, so we cannot infer whether the improved muscularity in CF is the result of increased physical activity. Establishing this association would be clinically important as qualitative indices of body composition such as fat-free mass have been linked with better respiratory outcomes and better quality of life [11, 26]. In this sense, the next line of research might be a randomised trial of a structured aerobic activity programme to adolescents with CF and establishing whether this intervention would lead to improvements in their aerobic capacity and whether an improved aerobic state would be associated with improved spirometric and clinical outcomes such as hospitalisation and reported quality of life. A short residential rehabilitation program consisting of supervised respiratory and nutritional treatment and daily physical activity for 3 weeks was associated with a significant increase in FFM in 34 patients with CF between 6 and 40 years old [27].

In our study, we report a lack of association between fat-free mass and the presence of chronic infection. Dufresne et al., similarly, did not find that inflammation could predict fat-free mass and quadriceps muscle strength in adults with CF [28]. Some other studies, however, have suggested that CF might be characterised by a generalised systemic myopathy that probably is the result of a spill-over of circulating pro-inflammatory cytokines [29]. In a group of 122 CF patients with a median age of 13 years, impaired peripheral muscularity was reported in the children and young people with chronic *Pseudomonas aeruginosa* infection compared to the CF subjects with less severe or no infection [30]. Van de Weert-van Leeuwen et al. also studied 149 adolescents with CF and reported that the response to exercise was decreased in a state of increased inflammation, possibly via a catabolic effect of circulating cytokines on the peripheral muscles [31].These conflicting results might be partially explained by population differences and the complexity of controlling for all potential contributors in a multifactorial disease such as CF.

Although our study population included adolescents with abnormal indices of airway obstruction (FEV_1_) and hyperinflation (FRC), these indices were not related to the fat-free mass. This implies that in our population, the mechanism of fat-free mass depletion might not involve airflow limitation and might be predominantly related to muscle disuse and deconditioning. This finding is in agreement with some previous studies that have reported that although inhaled bronchodilators can improve lung function indices such as FEV_1_, they have no effect on exercise tolerance [32].

Although only one participant in our study was receiving systemic corticosteroids at the time of study, corticosteroids are sometimes used in shorter or longer courses to treat inflammation in CF lung disease. The negative impact of these agents on peripheral muscle function has been well reported in numerous patient groups [33]. The term steroid myopathy refers to a myopathy with a symmetric distribution involving the proximal extremity muscles, associated with long-term muscle atrophy, notably with very minimal or no associated pain [34]. The possible cumulative effects of these agents on fat-free mass should also be considered in the clinical context.

Our study has strengths and some limitations. To our knowledge, we were the first to investigate the determinants of fat-free mass in stable adolescents with cystic fibrosis, and we highlighted the potential beneficial effect of exercise on maintaining skeletal muscularity and overall health in cystic fibrosis. The association of liver disease with impaired fat-free mass is also a novel finding in the adolescent CF population, which has not been previously reported. We should acknowledge as a limitation our limited population size, but our study focused only on adolescents and was conducted in a single CF centre, and thus, uniformity of care was guaranteed. We also conducted a comprehensive assessment of lung function, respiratory muscle function, body composition, and exercise capacity, and we were able to derive meaningful conclusions on the interactions of these parameters. Some previous papers have described that bioelectrical impedance measurements might underestimate the real fat-free mass in CF, suggesting that this method has limited applicability in the assessment of body composition in individual patients with CF [35]. In our study, however, we adjusted our fat-free mass measurements by correcting with an equation that was specific to our device and was derived by gold standard measurements made with dual energy X-ray absorptiometry. Future studies could include the evaluation of inflammatory indices such as tumour necrosis factor α and interleukin 6 to further elucidate the role of inflammation in CF myopathy.

In conclusion, we have demonstrated that in adolescents with CF, physical activity exerts a beneficial effect on fat-free mass, while, contrarily, CF-related liver disease negatively affects fat-free mass.

## Data Availability

Data is available from the corresponding author upon reasonable request.

## Abbreviations

ATS: American Thoracic Society
BMI: Body mass index
CF: Cystic fibrosis
ERS: European Respiratory Society
FEV_1_: Forced expiratory volume in 1 s
FFMI: Fat-free mass index
FRC: Functional residual capacity
IQR: Interquartile range
